# Retrospective Analysis of Therapeutic Modalities in Prosthetic Heart Valve Thrombosis: A 15-Year Single-Center Experience

**DOI:** 10.3390/medicina61091629

**Published:** 2025-09-09

**Authors:** Alper Uzunselvi, Serkan Yüksel, Muhammet Uyanik

**Affiliations:** 1 Department of Cardiology, Samsun Carsamba State Hospital, 55502 Samsun, Turkey; alpuzu05@gmail.com; 2 Department of Cardiology, Faculty of Medicine, Ondokuz Mayis University, 55139 Samsun, Turkey; serkan.yuksel@omu.edu.tr

**Keywords:** prosthetic valve thrombosis, mechanical heart valve, low-dose fibrinolysis, tissue plasminogen activator, NYHA functional class, treatment outcomes

## Abstract

*Background and Objectives*: Prosthetic valve thrombosis (PVT) represents a rare but critical complication after heart valve replacement surgery. This study aimed to evaluate patient characteristics, treatment modalities (medical vs. surgical), and clinical outcomes in patients with PVT over a 15-year period, with a particular focus on the impact of New York Heart Association (NYHA) functional class on mortality. *Materials and Methods*: We retrospectively analyzed 76 patients with confirmed PVT (54 mitral, 20 aortic, and 2 tricuspid; 97.4% mechanical) treated at a single tertiary center between 2005 and2020. The treatment comprised intravenous unfractionated heparin (UFH) alone (*n* = 29), low-dose tissue plasminogen activator (t-PA) (*n* = 27), or surgical re-operation (*n* = 20). Primary endpoints were treatment success, in-hospital mortality, and complications. *Results*: Overall, the treatment success was 60.5% (46/76) with a 25.0% (19/76) in-hospital mortality. UFH therapy achieved a 67.6% success with a 24.3% mortality. Low-dose t-PA demonstrated a 59.3% success with a significantly lower mortality (7.4%, *p* = 0.004). The surgery showed a 50% success with a 50% mortality. Patients in the NYHA class III-IV had markedly higher mortality (68.2% vs. 11.1%, *p* < 0.001) and lower treatment success (27.3% vs. 81.5%, *p* < 0.001) compared to the NYHA class I-II. A multivariate analysis revealed NYHA III–IV as the strongest predictor of mortality (OR 12.639, 95% CI: 1.905–83.849, *p* = 0.009). *Conclusions*: The low-dose t-PA (25 mg total dose) therapy showed the lowest mortality among treatment modalities. The NYHA functional class emerged as the most significant predictor of outcomes, with the class III-IV patients having >12-fold increased mortality risk. These findings support early intervention and suggest that t-PA is a viable first-line option in selected patients.

## 1. Introduction

Prosthetic heart valve thrombosis (PVT) represents a rare but critical complication occurring after heart valve replacement surgery, characterized primarily by thrombus or pannus formation. Pannus develops gradually, typically presenting as granulation tissue accumulation, whereas thrombus formation occurs suddenly, often accompanied by significant congestive symptoms. Frequently, these conditions coexist and exacerbate each other, complicating clinical management [1,2].

Echocardiographic assessment is essential in diagnosing and managing PVT. Typical echocardiographic findings include elevated transvalvular gradients, reduced or restricted leaflet mobility, presence of thrombotic masses on valve leaflets or hinges, and occasionally valve obstruction. The thrombus size, location, mobility, and presence of associated pannus tissue can be clearly visualized using transthoracic (TTE) or transesophageal echocardiography (TEE), with TEE being particularly valuable in cases where thrombus is not readily apparent on TTE due to suboptimal imaging windows or the small size of thrombus. Advanced echocardiographic modalities, including three-dimensional echocardiography (3D-echo), provide superior spatial resolution, allowing a detailed morphological characterization of the thrombus, an accurate measurement of thrombus size, and an assessment of its precise localization and impact on valve mechanics. Such detailed imaging assessments significantly influence clinical decision-making by guiding therapeutic strategy selection (medical vs. surgical treatment) and aiding in prognosis prediction. Recent studies emphasize the added diagnostic value and clinical utility of integrating advanced imaging techniques such as 3D echocardiography and computed tomography (CT) in the management of PVT, particularly in ambiguous or complicated cases [3].

The clinical presentation of PVT varies considerably, ranging from asymptomatic incidental findings to severe presentations, including acute dyspnoea, systemic embolism, or cardiogenic shock. The obstructive thrombosis typically results in hemodynamic compromise, evident through elevated valve gradients, reduced valve orifice area, and symptoms indicative of decreased cardiac output and heart failure [4].

Vitamin K antagonists (VKAs) remain the only approved oral anticoagulants for patients with prosthetic heart valves. Even with the VKA therapy, the annual risk of thromboembolic complications remains at approximately 1–2%, significantly rising with an inadequate anticoagulation or its absence [5,6]. The PVT incident in mechanical prosthetic valves ranges from 0.5% to 8% for mitral and aortic valves, and it is notably higher, around 20%, for tricuspid valves, suggesting that inadequate anticoagulant therapy remains the predominant factor contributing to PVT [7].

Treatment options for PVT include intensified anticoagulation, thrombolytic therapy (TT), and surgical intervention. Historically, surgery was considered the primary treatment until the early 1990s. Recently, thrombolytic therapy (TT) has become an increasingly accepted alternative, particularly for patients with obstructive thrombus [7,8]. However, a robust evidence from randomized controlled trials comparing these treatments remains scarce, prompting the ongoing debate regarding optimal initial management strategies [9].

Guidelines of the European Society of Cardiology (ESC) advocate surgical intervention primarily for patients who are critically ill, reserving thrombolysis for individuals at high surgical risk or with the right-sided valve thrombosis. Treatment decisions are significantly influenced by factors such as the degree of valve obstruction, valve location, and clinical severity [10].

This study retrospectively evaluates demographic characteristics, systemic diseases, pre-existing cardiac conditions, presenting symptoms, functional capacity, echocardiographic findings, laboratory parameters, treatment modalities, complications, and in-hospital mortality outcomes of 76 patients diagnosed with prosthetic heart valve thrombosis at Ondokuz Mayıs University’s Faculty of Medicine Hospital over a 15-year period. Through examining real-world data, this research aims to offer insights to enhance clinical decision-making and optimize management strategies for prosthetic valve thrombosis cases.

## 2. Materials and Methods

This retrospective study was conducted on 76 patients diagnosed and treated for prosthetic heart valve thrombosis (PVT) at Ondokuz Mayıs University’s Faculty of Medicine Hospital between 1 January 2005, and 31 December 2020.

**Figure 1:**
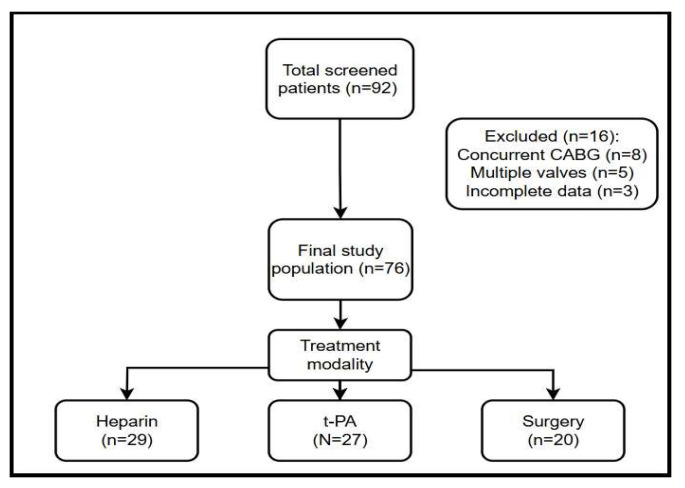
Enrollment process diagram.

The inclusion criteria in this study were adult patients (≥18 years old) who had undergone a heart valve replacement surgery with either mechanical or bioprosthetic valves and were subsequently diagnosed with PVT. The exclusion criteria were patients who had undergone concurrent procedures such as coronary artery bypass grafting combined with a valve replacement, multiple valve replacements, or other significant cardiovascular procedures during the follow-up (Figure 1).

Patients data were collected retrospectively from electronic medical records at the cardiology and cardiovascular surgery departments. The collected data included demographic information, comorbidities, pregnancy status, presenting symptoms, physical examination findings, New York Heart Association (NYHA) functional classification, echocardiographic parameters, laboratory results, valve age (time since implantation to thrombosis event), treatment strategies employed, complications, and in-hospital mortality.

The diagnosis of PVT was established based on the clinical presentation along with diagnostic imaging studies, including transthoracic echocardiography (TTE), transesophageal echocardiography (TEE), and cinefluoroscopy. Thrombosis was classified as obstructive based on elevated transvalvular gradients detected by echocardiography or reduced/fixed leaflet mobility observed via cinefluoroscopy [1,2].

Echocardiographic assessments were conducted by experienced cardiologists. The presence of valve obstruction was identified using maximum and mean transvalvular gradient criteria: maximum gradient > 50 mmHg and mean gradient > 35 mmHg for aortic prostheses; mean gradient > 10 mmHg for mitral prostheses; and mean gradient > 6 mmHg for tricuspid prostheses. The thrombus size was defined as large if the diameter was ≥10 mm or the cross-sectional area exceeded 0.8 cm^2^ [7].

### 2.1. Treatment Strategies

Treatment strategies were decided by the attending physicians and consisted of: (1) the intensification of anticoagulation therapy using intravenous unfractionated heparin (UFH) followed by oral warfarin; (2) the thrombolytic therapy (TT) using a tissue plasminogen activator (t-PA); and (3) the surgical re-operation (surgery for the valve replacement) [8,9].

Intravenous UFH was administered with an initial bolus of 80 units/kg followed by continuous infusion of 18 units/kg/hour, adjusted to maintain activated partial thromboplastin time (aPTT) 1.5–2.5 times control. The low-dose t-PA protocol consisted of a 25 mg total dose administered as 6 mg bolus followed by a 19 mg infusion over 6 hour for patients < 75 kg, or 10 mg bolus followed by a 15 mg infusion for patients ≥ 75 kg. The criteria for thrombolytic therapy included a recent valve implantation (≤2 months prior), a small thrombus (<8 mm^2^), and patients with the NYHA functional class I-II who did not meet the surgical intervention criteria. Success was defined as a significant clinical improvement, a reduction of transvalvular gradients, >50% decrease in thrombus diameter, and an absence of major complications such as stroke, intracranial hemorrhage (ICH), or death. Failure was defined as the occurrence of death, major complications, or the need for a surgical intervention after an unsuccessful thrombolysis [7,11].

Indications for a surgical re-operation included a large thrombus (>8 mm^2^), a pannus formation, a recurrent thrombosis despite the thrombolytic therapy, contraindications to thrombolysis, and a severe clinical presentation (NYHA III–IV functional status) [12,13]. In the selected hemodynamically stable patients with a non-obstructive thrombus and a high surgical risk, the initial treatment with intravenous UFH may still be considered [14].

### 2.2. Statistical Analysis

Statistical analyses were conducted using the IBM SPSS Statistics software version 23.0. The normal distribution was evaluated using the Shapiro–Wilk test, as it provides great power for small sample sizes. When data violated the normality assumptions, the median and interquartile range (IQR) were reported instead of the mean ± standard deviation. Categorical variables were expressed as frequencies and percentages.

For comparisons between two groups, independent *t*-tests were used for normally distributed continuous variables, while the Mann–Whitney U tests were employed for non-normally distributed data. When comparing three or more groups (e.g., treatment modalities), one-way ANOVA was used for normally distributed data and the Kruskal–Wallis test for non-normally distributed data. Post-hoc analyses with the Bonferroni correction were performed when significant differences were detected. The treatment success and mortality rates were compared across the treatment groups using Pearson’s chi-square test, or Fisher’s exact test when expected cell counts were smaller than five. A two-tailed *p*-value < 0.05 was considered statistically significant.

## 3. Results

### 3.1. Baseline Demographics and Clinical Characteristics

Seventy-six patients with confirmed prosthetic valve thrombosis were analyzed (Table 1). The cohort comprised middle-aged patients (mean age 53 ± 17 years) with female predominance (63.2%, *n* = 48), including 11 pregnant women (22.9% of females).

Only 21.1% (*n* = 16) of the patients had the therapeutic anticoagulation (INR ≥ 2.5), while 78.9% exhibited subtherapeutic levels (median INR 1.60, range 0.99–5.41). Mechanical valves comprised 97.4% (*n* = 74) vs. 2.6% bioprosthetic (*n* = 2). The mitral position predominated (71.1%, *n* = 54), followed by aortic (26.3%, *n* = 20) and tricuspid (2.6%, *n* = 2). The median prosthesis age was 7.5 years (with a range of 0.1–28). Comorbidities included atrial fibrillation (39.5%, *n* = 30), hypertension (36.8%, *n* = 28), and diabetes mellitus (19.7%, *n* = 15). The clinical presentations comprised acute heart failure (48.7%, *n* = 37), cerebrovascular events (22.4%, *n* = 17), peripheral embolism (11.8%, *n* = 9), and asymptomatic cases (9.2%, *n* = 7). The functional assessment showed 71.1% (*n* = 54) in the NYHA class I-II vs. 28.9% (*n* = 22) in class III-IV. The mean left ventricular ejection fraction was 51.7 ± 9.6%. The echocardiographic evaluation revealed elevated transvalvular gradients (Table 2). Mitral prostheses showed median maximum and mean gradients of 20 mmHg (range 9–49) and 10 mmHg (range 3.5–28), respectively. Aortic prostheses demonstrated median maximum and mean gradients of 43 mmHg (range 14–120) and 27 mmHg (range 8–80). The median thrombus diameter was 10 mm (range 2–47), with 55.3% having thrombi ≥ 10 mm. The median surface area was 70 mm^2^ (range 3–1457), with 47.4% exceeding 80 mm^2^. The combined criteria classified 71.1% as having a large thrombus burden.

### 3.2. Treatment Modalities and Success Rates

The treatment comprised a UFH therapy (38.2%, *n* = 29), the t-PA thrombolysis (35.5%, *n* = 27), and the surgical re-operation (26.3%, *n* = 20) (Table 3). Overall, a treatment success was achieved in 46 patients (60.5%), while in-hospital mortality occurred in 19 patients (25.0%). The treatment success rate was 69.0% in the heparin group, 59.3% in the t-PA group, and 50.0% in the surgery group (*p* = 0.405, Table 3). The mortality rate was 24.1% in the heparin group, 7.4% in the t-PA group, and 50.0% in the surgery group (*p* = 0.004, Table 3). The mortality rate was significantly higher in the surgery group compared with the other treatment groups. The NYHA classes demonstrated dramatic differences in the mortality rates: class III-IV 68.2% vs. class I-II 11.1% (*p* < 0.001). The obstructive thrombus showed higher mortality (38.1%) vs. non-obstructive (14.7%, *p* = 0.023). The treatment success also varied by the NYHA class: I-II and III-IV achieved a 81.5% and a 27.3% success, respectively (*p* < 0.001).

### 3.3. Complication Analysis

The complication events occurred in 42.1% (*n* = 32) of the patients (Table 4). Complications are defined as any embolic event, bleeding episode, prosthesis dysfunction, infective endocarditis, or wound infection occurring during hospitalization [15].

The NYHA III–IV group had significantly higher complication rates (81.8%, *n* = 18/22) vs. the NYHA I–II group (25.9%**,** *n* = 14/54, *p* < 0.001). The treatment-specific complication rates were: UFH 31.0% (*n* = 9/29), t-PA 18.5% (*n* = 5/27), and surgery 40.0% (*n* = 8/20, *p* = 0.262).

The embolic complications occurred in 10.5% (*n* = 8) of the patients, with major embolic events in 5.3% (*n* = 4), including stroke and peripheral arterial embolism. A minor bleeding occurred in 10.5% (*n* = 8) of the patients, while a major bleeding, requiring transfusion, occurred in 6.6% (*n* = 5). The intracranial hemorrhage developed in 3.9% (*n* = 3) of all patients: 3.4% (*n* = 1/29) of UFH-treated and 7.4% (*n* = 2/27) of t-PA-treated patients.

The obstructive thrombus was associated with higher complication rates (54.8%, *n* = 23/42) vs. the non-obstructive thrombus (26.5%, *n* = 9/34, *p* = 0.013). Among the nine patients who initially presented with a peripheral embolism, four (44.4%) experienced additional complications during treatment, representing a significantly higher rate than other presentation groups (*p* = 0.007).

The multivariate logistic regression analysis identified independent mortality predictors (Table 5). The model achieved Nagelkerke R^2^ = 0.59 with 71.4% sensitivity and 93.3% specificity. The NYHA functional class III-IV was the sole independent predictor (OR: 12.64, 95% CI: 1.91–83.85, *p* = 0.009). Other variables including age (*p* = 0.646), obstructive thrombus (*p* = 0.227), t-PA treatment (*p* = 0.207), surgery (*p* = 0.263), and INR (*p* = 0.796) were not statistically significant.

## 4. Discussion

The prosthetic valve thrombosis remains one of the most challenging complications in cardiovascular medicine, with management decisions often requiring careful balance between therapeutic efficacy and risk-benefit ratios. Our 15-year single-center experience provides valuable insights into real-world outcomes and highlights critical factors influencing patient prognosis.

### 4.1. Treatment Efficacy and Comparative Outcomes

In our cohort, the overall treatment success was 60.5% and the in-hospital mortality was 25.0%. Thrombolysis achieved a mortality of 7.4%, which is lower than UFH (24.1%) and surgery (50.0%), but it is higher than 2.8%, the rate has been reported in the TROIA study—likely reflecting differences in case mix and real-world acuity. These findings support considering low-dose t-PA in appropriately selected, hemodynamically stable patients, while acknowledging center- and patient-level variability [7,16,17].

The superior mortality profile of thrombolytic therapy compared to surgical intervention (50% mortality) in our series deserves particular attention. While surgery has traditionally been considered the gold standard for PVT management, our findings support the growing body of evidence favoring the thrombolytic therapy as a first-line treatment in appropriately selected patients. The 50% surgical mortality in our cohort, though higher than some reported series, reflects the real-world challenges faced in tertiary care centers managing patients with a critical illness who are often deemed unsuitable for the thrombolytic therapy.

Interestingly, the UFH therapy achieved the highest success rate (67.6%) with a moderate mortality (24.3%), suggesting that in carefully selected patients with small, non-obstructive thrombi, an intensive anticoagulation alone may be sufficient. This finding has important implications for clinical practice, particularly in patients with contraindications to the thrombolytic therapy or a high surgical risk.

### 4.2. The NYHA Functional Class as a Prognostic Determinant

The NYHA functional class emerged as the dominant prognostic marker: class III–IV was associated with a substantially lower treatment success and a markedly higher in-hospital mortality compared with class I–II. This underscores the clinical value of early recognition and intervention before the advanced symptomatic deterioration. While the adjusted effect size carried wide confidence intervals due to the sample size, the direction and magnitude were consistent with the prior reports.

### 4.3. Thrombus Characteristics and Clinical Decision-Making

The obstructive thrombus and larger thrombus burden were associated with higher complication and mortality rates, in line with previous observational studies [9,18]. Although current guidelines recommend surgery for large thrombi (>0.8 cm^2^) or pannus formation [10,12], our findings suggest that the low-dose thrombolysis may still be considered in carefully selected, hemodynamically stable patients with a high surgical risk. These results highlight the need for individualized decision-making that accounts for both anatomical factors and the overall clinical status.

### 4.4. Anticoagulation Management and Prevention

A concerning finding in our study was that 78.9% of patients had subtherapeutic INR levels at presentation, highlighting the ongoing challenge of an optimal anticoagulation management in prosthetic valve patients. This observation underscores the critical importance of patient education, regular monitoring, and potentially novel anticoagulation strategies, though direct oral anticoagulants remain contraindicated in mechanical valve patients.

The high prevalence of inadequate anticoagulation in our cohort reflects real-world challenges including patient non-compliance, drug interactions, dietary factors, and healthcare system limitations. This finding emphasizes the need for comprehensive anticoagulation management programs and potentially point-of-care INR monitoring in high-risk patients.

### 4.5. Pregnancy and Special Populations

The presence of 22.9% pregnant patients among females in our cohort highlights the unique challenges of managing PVT in pregnancy. Pregnancy-related hypercoagulability, combined with the complexity of anticoagulation management during gestation, creates a particularly high-risk scenario requiring multidisciplinary care involving cardiologists, obstetricians, and hematologists.

### 4.6. Implications for Clinical Practice

Our findings have several important implications for clinical practice. First, the superior mortality profile of thrombolytic therapy supports its consideration as the first-line treatment in hemodynamically stable patients without absolute contraindications. Second, the critical importance of the NYHA functional class suggests that the clinical assessment should heavily weight functional status in treatment decision-making. Third, the poor outcomes in advanced heart failure patients emphasize the need for early detection and intervention strategies. Additionally, in the select cases with small, non-obstructive thrombi, particularly when the surgical risk is high or the thrombolysis contraindicated, the intensified anticoagulation therapy alone may represent a sufficient initial therapeutic strategy [12].

The development of institutional protocols for managing PVT, incorporating risk stratification based on the NYHA class, thrombus characteristics, and patient comorbidities may improve outcomes. We suggest that a multidisciplinary approach involving cardiologists, cardiac surgeons, and hematologists is essential for optimal patient care.

### 4.7. Future Directions

Future research should focus on developing more sophisticated risk prediction models that incorporate not only anatomical factors but also functional status, biomarkers, and advanced imaging parameters. Specifically, incorporating granular imaging data obtained from three-dimensional echocardiography (3D-echo) and cardiac computed tomography (CT) would substantially enhance thrombus characterization, including morphology, mobility, and precise localization. Such detailed imaging could significantly improve therapeutic decision-making, patient-specific risk stratification, and overall treatment outcomes.

Furthermore, exploring the role of biomarkers—such as cardiac troponins, natriuretic peptides (e.g., NT-proBNP), or inflammatory markers—and additional clinical risk stratification tools alongside the NYHA functional class may help to predict outcomes more robustly. Integrating these biochemical and clinical parameters could lead to better identification of high-risk patients, allowing clinicians to personalize therapeutic strategies and potentially reduce adverse outcomes.

Additionally, the development of novel thrombolytic regimens with improved efficacy and safety profiles, as well as mechanical thrombectomy devices specifically designed for prosthetic valves, may expand treatment options and improve prognosis in this challenging patient population.

### 4.8. Limitations

Our study is subject to several important limitations inherent in its retrospective design. The selection biases may have influenced the treatment allocation, as the sicker patients were more likely to undergo surgical intervention while the stable patients received a medical therapy. The lack of standardized treatment protocols across the 15-year study period may have introduced variability in management approaches, though this also reflects the real-world clinical practice.

The relatively small sample size, particularly for subgroup analyses, may limit the generalization of our findings. Additionally, the single-center design may not reflect outcomes achievable in different healthcare settings with varying resources and expertise levels. Another critical limitation is the lack of long-term follow-up data. Our analysis was restricted to the in-hospital outcomes, which limit our ability to evaluate the durability of treatment success, recurrence rates, valve dysfunction, and other late complications such as thromboembolic events, hemorrhage, or mortality. Future prospective studies with longer follow-up durations are essential to address these gaps in knowledge.

Despite these limitations, the extended study period and the real-world clinical setting enhance the external validity of our findings, providing important insights into daily clinical practice that complement data derived from randomized controlled trials.

## 5. Conclusions

Early diagnosis and timely intervention are pivotal in PVT, with functional status (NYHA class) strongly informing the prognosis. In our experience, low-dose t-PA showed the most favorable in-hospital mortality among strategies for appropriately selected, stable patients, whereas surgery carried higher risk in a sicker subset. The intensified anticoagulation may suffice in carefully chosen cases with small, non-obstructive thrombi. An individualized, team-based decision-making that integrates the clinical severity and anatomical features remains essential.

## Figures and Tables

**Table 1:** Baseline Demographic, Clinical Characteristics and Comorbidities of Patients with Prosthetic Valve Thrombosis.

Parameter	Overall (*n* = 76)	UFH (*n* = 29)	t-PA (*n* = 27)	Surgery (*n* = 20)	*p*-Value
Demographics					
Age, years (mean ± SD)	53 ± 17	52 ± 18	50 ± 15	57 ± 17	0.47
Female, n (%)	48 (63.2)	19 (65.5)	18 (66.7)	11 (55.0)	0.72
Pregnancy †, n (%)	11/48 (22.9)	5/19 (26.3)	4/18 (22.2)	2/11 (18.2)	0.91
Comorbidities, n (%)					
Atrial fibrillation	30 (39.5)	11 (37.9)	10 (37.0)	9 (45.0)	0.88
Hypertension	28 (36.8)	10 (34.5)	9 (33.3)	9 (45.0)	0.77
Diabetes mellitus	15 (19.7)	6 (20.7)	5 (18.5)	4 (20.0)	0.98
Laboratory					
INR at presentation, (median, range)	1.60 (0.99–5.41)	1.55 (1.0–3.5)	1.60 (1.1–5.4)	1.65 (1.2–4.5)	0.62
INR ≥ 2.5, n (%)	16 (21.1)	5 (17.2)	6 (22.2)	5 (25.0)	0.81
Valve type, n (%)					
Mechanical valve	74 (97.4)	28 (96.6)	27 (100)	19 (95.0)	0.55
Bioprosthetic valve	2 (2.6)	1 (3.4)	0 (0)	1 (5.0)	-
Valve position, n (%)					
Mitral position	54 (71.1)	20 (69.0)	20 (74.1)	14 (70.0)	0.92
Aortic position	20 (26.3)	8 (27.6)	6 (22.2)	6 (30.0)	0.84
Tricuspid position	2 (2.6)	1 (3.4)	1 (3.7)	0 (0)	0.66
Prosthesis age, years (median, range)	7.5 (0.1–28)	7.3 (0.2–25)	7.6 (0.3–26)	7.8 (0.1–28)	0.89
Clinical presentation, n (%)					
Acute heart failure	37 (48.7)	12 (41.4)	13 (48.1)	12 (60.0)	0.56
Cerebrovascular event	17 (22.4)	6 (20.7)	6 (22.2)	5 (25.0)	0.94
Peripheral embolism	9 (11.8)	3 (10.3)	3 (11.1)	3 (15.0)	0.88
Asymptomatic	7 (9.2)	3 (10.3)	2 (7.4)	2 (10.0)	0.93
NYHA I–II	54 (71.1)	20 (69.0)	20 (74.1)	14 (70.0)	0.93
NYHA III–IV	22 (28.9)	9 (31.0)	7 (25.9)	6 (30.0)	0.93

INR, international normalized ratio; NYHA, New York Heart Association; SD, standard deviation. Continuous variables: normally distributed data presented as mean ± SD; non-normally distributed data presented as median (Min–Max). ^†^ Percentage among female patients.

**Table 2:** Echocardiographic and Thrombus Parameters.

Parameter, mmHg (Median, Range)	Overall (*n* = 76)	UFH (*n* = 29)	t-PA (*n* = 27)	Surgery (*n* = 20)	*p*-Value
Mitral max gradient,	20 (9–49)	18 (9–40)	20 (10–45)	22 (12–49)	0.41
Mitral mean gradient, mmHg (median, range)	10 (3.5–28)	9 (3.5–20)	10 (4–24)	11 (4–28)	0.39
Aortic max gradient, mmHg (median, range)	43 (14–120)	40 (14–100)	42 (15–110)	45 (20–120)	0.55
Aortic mean gradient, mmHg (median, range)	27 (8–80)	25 (8–75)	26 (10–78)	30 (15–80)	0.58
Thrombus diameter, mm (median, range)	10 (2–47)	9 (2–25)	10 (3–30)	12 (5–47)	0.44
Thrombus surface area, mm^2^ (median, range)	70 (3–1457)	65 (5–850)	70 (10–900)	85 (15–1457)	0.48
Left ventricular EF, % (mean ± SD)	51.7 ± 9.6	52.5 ± 9.3	51.2 ± 10.1	51.0 ± 9.5	0.83

EF, ejection fraction; SD, standard deviation. Continuous echo and thrombus parameters are skewed; values reported as median (Min–Max).

**Table 3:** Success and Mortality Rates of the Treatment for Different Groups.

Outcome	UFH (*n* = 29)	t-PA (*n* = 27)	Surgery (*n* = 20)	*p*-Value
Treatment success, *n* (%)	20 (69.0%)	16 (59.3%)	10 (50.0%)	0.405
Mortality, *n* (%)	7 (24.1%)	2 (7.4%)	10 (50.0%)	0.004

t-PA, tissue plasminogen activator. Values are expressed as *n* (%). *p*-values represent comparisons between treatment groups using chi-square test (or Fisher’s exact test where appropriate) for both success and mortality.

**Table 4:** Complication Profiles by Modality.

Complication Type	UFH (*n* = 29)	t-PA (*n* = 27)	Surgery (*n* = 20)	Overall (*n* = 76)	*p*-Value
Embolic Events					
Major embolic events	2 (6.9%)	1 (3.7%)	1 (5.0%)	4 (5.3%)	0.865
Minor embolic events	1 (3.4%)	2 (7.4%)	0 (0.0%)	3 (3.9%)	0.429
Total embolic events	3 (10.3%)	3 (11.1%)	1 (5.0%)	8 (10.5%)	0.727
Bleeding Events					
Major bleeding	2 (6.9%)	0 (0.0%)	3 (15.0%)	5 (6.6%)	0.122
Minor bleeding	3 (10.3%)	2 (7.4%)	2 (10.0%)	8 (10.5%)	0.921
Intracranial hemorrhage	1 (3.4%)	2 (7.4%)	0 (0.0%)	3 (3.9%)	0.372
Other Complications					
Prosthesis dysfunction	1 (3.4%)	0 (0.0%)	0 (0.0%)	1 (1.3%)	0.440
Infective endocarditis	0 (0.0%)	0 (0.0%)	1 (5.0%)	1 (1.3%)	0.242
Wound infection	0 (0.0%)	0 (0.0%)	1 (5.0%)	1 (1.3%)	0.242
Total patients with complications ‡	9 (31.0%)	5 (18.5%)	8 (40.0%)	32 (42.1%)	0.262

t-PA, tissue plasminogen activator. Values are expressed as *n* (%). *p*-values represent comparisons between UFH, t-PA, and surgery groups using chi-square or Fisher’s exact test ‡ Individual patients could experience multiple types of complications.

**Table 5:** Multivariate Logistic Regression for In-Hospital Mortality.

Variable	OR	95% CI	*p*-Value
NYHA III–IV vs. I–II	12.64	1.91–83.85	0.009
Obstructive thrombus	5.64	0.34–93.58	0.227
t-PA treatment	0.21	0.02–2.37	0.207
Surgery	3.10	0.43–22.46	0.263
Age (per year)	1.02	0.95–1.08	0.646
INR at presentation	1.36	0.13–14.28	0.796

CI, confidence interval; INR, international normalized ratio; NYHA, New York Heart Association; OR, odds ratio; t-PA, tissue plasminogen activator. Model: Nagelkerke R^2^ = 0.59; sensitivity 71.4%; specificity 93.3%.

## Data Availability

The data underlying this article cannot be shared publicly due to patient privacy concerns and institutional regulations regarding retrospective clinical data. The data contain potentially identifying patient information from electronic medical records at Ondokuz Mayıs University Faculty of Medicine Hospital.

## Abbreviations

The following abbreviations are used in this manuscript:

EF	Ejection Fraction
CI	Confidence Interval
ESC	European Society of Cardiology
ICH	Intracranial Hemorrhage
INR	International Normalised Ratio
NYHA	New York Heart Association
PVT	Prosthetic Valve Thrombosis
TEE	Transesophageal Echocardiography
TTE	Transtoragic Echocardiography
t-PA	tissue Plasminogen Activator
UFH	Unfractionated Heparin
